# Coli Surface Antigen 26 Acts as an Adherence Determinant of Enterotoxigenic *Escherichia coli* and Is Cross-Recognized by Anti-CS20 Antibodies

**DOI:** 10.3389/fmicb.2018.02463

**Published:** 2018-10-16

**Authors:** Leandro Cádiz, Alexia Torres, Raúl Valdés, Gabriel Vera, Daniela Gutiérrez, Myron M. Levine, David A. Montero, Miguel O’Ryan, David A. Rasko, O. Colin Stine, Roberto Vidal, Felipe Del Canto

**Affiliations:** ^1^Programa de Microbiología y Micología, Instituto de Ciencias Biomédicas, Facultad de Medicina, Universidad de Chile, Santiago, Chile; ^2^Facultad de Química y Biología, Universidad de Santiago de Chile, Santiago, Chile; ^3^Facultad de Ciencias Químicas y Farmacéuticas, Universidad de Chile, Santiago, Chile; ^4^Center for Vaccine Development and Global Health, Department of Microbiology and Immunology, University of Maryland School of Medicine, Baltimore, MD, United States; ^5^Instituto Milenio de Inmunología e Inmunoterapia, Facultad de Medicina, Universidad de Chile, Santiago, Chile; ^6^Institute for Genome Sciences, Department of Microbiology and Immunology, University of Maryland School of Medicine, Baltimore, MD, United States; ^7^Department of Epidemiology and Public Health, University of Maryland School of Medicine, Baltimore, MD, United States

**Keywords:** ETEC, virulence factors, bacterial adherence, pilus, chaperone-usher pili, colonization factors, CS26

## Abstract

The coli surface antigen 26 (CS26) of enterotoxigenic *Escherichia coli* (ETEC) had been described as a putative adhesive pilus based on the partial sequence of the *crsH* gene, detected in isolates from children with diarrhea in Egypt. However, its production and activity as adherence determinant has not been experimentally addressed. The *crsH* was identified as a homolog of genes encoding structural subunits of ETEC colonization factors (CFs) CS12, CS18, and CS20. These CFs, along with the recently discovered CS30, belong to the γ_2_ family of pili assembled by the chaperone-usher pathway (CU pili). Further, the complete CS26 locus, *crsHBCDEFG*, was described in an O141 ETEC strain (ETEC 100664) obtained from a diarrhea case in The Gambia, during the Global Enterics Multicenter Study. Here, we report that CS26 is a pilus of ∼10 nm in diameter, with the capacity to increase the cell adherence of the non-pathogenic strain *E. coli* DH10B. As for other related pili, production of CS26 seems to be regulated by phase variation. Deletion of *crsHBCDEFG* in ETEC 100664 significantly decreased its adherence capacity, which was recovered by *in trans* complementation. Furthermore, CrsH was cross-recognized by polyclonal antibodies directed against the major structural subunit of CS20, CsnA, as determined by Western blotting and immunogold labeling. ETEC CS26+ strains were found to harbor the heat-labile enterotoxin only, within three different sequence types of phylogroups A and B1, the latter suggesting acquisition through independent events of horizontal transfer. Overall, our results demonstrate that CS26 is an adhesive pilus of human ETEC. In addition, cross-reactivity with anti-CsnA antibodies indicate presence of common epitopes in γ_2_-CFs.

## Introduction

Enterotoxigenic *Escherichia coli* (ETEC) causes diarrhea in humans by secreting heat-labile toxin (LT) and/or heat-stable toxins (STh and STp) (Gomes et al., 2016). ETEC infections are responsible for millions of diarrhea cases worldwide and about 60,000 deaths every year, mainly in children under 5 years in developing countries (Khalil, 2017). In addition, ETEC are the main cause of traveler’s diarrhea. Despite a progressive decline in ETEC associated deaths, there is a consensus that a vaccine against ETEC is needed (Hosangadi et al., 2018).

ETEC are a diverse group of pathogenic *E. coli* which produce a diverse set of virulence factors. Given that ETEC must adhere to epithelial cells to optimally induce a toxigenic effect, structures determining attachment are eligible targets for vaccine development (Dorsey et al., 2006; O’Ryan et al., 2015). The colonization factors (CFs), 23 functionally characterized proteinaceous surface pili, are the classical ETEC adherence determinants of which 18 are assembled by the chaperone-usher pathway (CU pili), a common mechanism to construct pili at surface of Gram-negative bacteria (Madhavan and Sakellaris, 2015; Del Canto et al., 2017). Chaperones are periplasmic proteins that bind and fold pilus structural subunits for assembly, which occurs at the usher, an outer membrane pore-forming platform. Pili can be formed by one or more structural subunits. The most abundant and repetitive subunit in the pilus is known as the major structural subunit while others are considered as minor structural subunits (Busch and Waksman, 2012).

Bacterial pili have been classified according to several different schemes. Based on the usher sequence, nine families have been defined: α, β, γ_1_, γ_2_, γ_3_, γ_4_, κ, π, and σ (Nuccio and Bäumler, 2007). The most common ETEC CU-CFs are found in families α (CFA/I, CS1, CS2, CS4, CS5, CS7, CS14, CS17, and CS19) and γ_3_ (CS3, CS6) (Madhavan and Sakellaris, 2015). The γ_2_ family included three CFs, CS12, CS18, and CS20, which are not frequently detected (Isidean et al., 2011; Madhavan and Sakellaris, 2015); however, genetic analyses of CF-negative strains are adding more representatives of γ_2_-CFs to the list. In fact, the most recent CF identified, CS30, was found to be similar to CS18 and CS20 (von Mentzer et al., 2017). Additionally, degenerate-PCR had allowed identification of segments of three genes encoding putative major structural subunits of γ_2_-CFs; *crsH, cmaH*, and *cnmH*, which were designated as the putative CFs CS26, CS27, and CS28 (Nada et al., 2011). Later, their full loci were identified in ETEC isolates obtained from diarrhea cases and six other putative γ_2_-CF loci were described (Del Canto et al., 2017; Sahl et al., 2017). Thus, ETEC strains negative in the detection of CFs seem to produce mainly γ_2_-CU pili. The data from comparative genomic analysis which led to these findings, is highly valuable but requires validation with experimental assessment in order to prove functional predictions. Here, we report the functional characterization of CS26.

## Materials and Methods

### Strains and DNA Vectors

Strains and DNA vectors used in this work are listed in **Table 1**. Strains were grown on Lysogeny broth (LB) or LB-agar containing ampicillin (100 μg/mL) chloramphenicol (12.5 or 30 μg/mL), kanamycin (50 μg/mL) or arabinose (0.01%) as required, at 32 or 37°C.

**Table 1:** Strains and plasmids used in this study.

Strain	Features	Reference
ETEC 100664	Enterotoxigenic *Escherichia coli* isolated from a child with diarrhea in The Gambia during the Global Enterics Multicenter Study (GEMS). Serogroup O141, LT+, CS26+ (*crsHBCDEFG*), Sequence type-165.	Kotloff et al., 2013; Del Canto et al., 2017
ETEC 100664 Δ*crs*	ETEC 100664 lacking *crsHBCDEFG* (CS26-).	Del Canto et al., 2017
ETEC 100664 Δ*crs*/*crs*-SV	ETEC 100664 Δ*crs* complemented *in trans* with pEZ-BAC/*crsHBCDEFG* (CS26+).	This work.
*E. coli* DH10B	*E. coli* K-12 derivate, genotype *F^-^ mcrA*Δ *(mrr-hsdRMS-mcrBC)*ϕ*80dlacZ*Δ*M15*Δ*lacX74 endA1 recA1 araD139*Δ *(ara,leu)7697 galU galK rpsL (StrR) nupG (attL araC-PBAD-trfA250 bla attR)*λ. This strain harbors the *trfA* gene, which allows to increase the copy number of pEZ-BAC by adding L-arabinose to the culture medium.	Lucigene (CopyRight^®^ v2.0 BAC Cloning Kits).

**DNA vectors**	**Features**	**Reference**

pEZ-BAC	*ori2, repE, IncC* - origin of replication (single copy); *oriV* – inducible origin of replication; *par A,B,C*- partition genes; *Cm*^R^- chloramphenicol resistance gene; *cosN* - lambda packaging signal; T-CloneSmart transcription terminators; *lacZ*, alpha peptide portion of the beta galactosidase gene.	Lucigene (CopyRight^®^ v2.0 BAC Cloning Kits).
pEZ-BAC/*crs*-SV	pEZ-BAC containing *crsHBCDEFG* locus cloned at the BamHI site.	This work.
pEZ-BAC/*crs*-LV	pEZ-BAC containing *crsSTHBCDEFG* locus cloned at the BamHI site.	This work.
pSIM6	Plasmid encoding the λ Red recombinase system.	Sharan et al., 2009
pCP20	Plasmid encoding the yeast Flp recombinase. Used to remove antibiotic resistance gene from the mutant strain.	Datsenko and Wanner, 2000

### Cloning of the *crs* Locus

**FIGURE 1:**
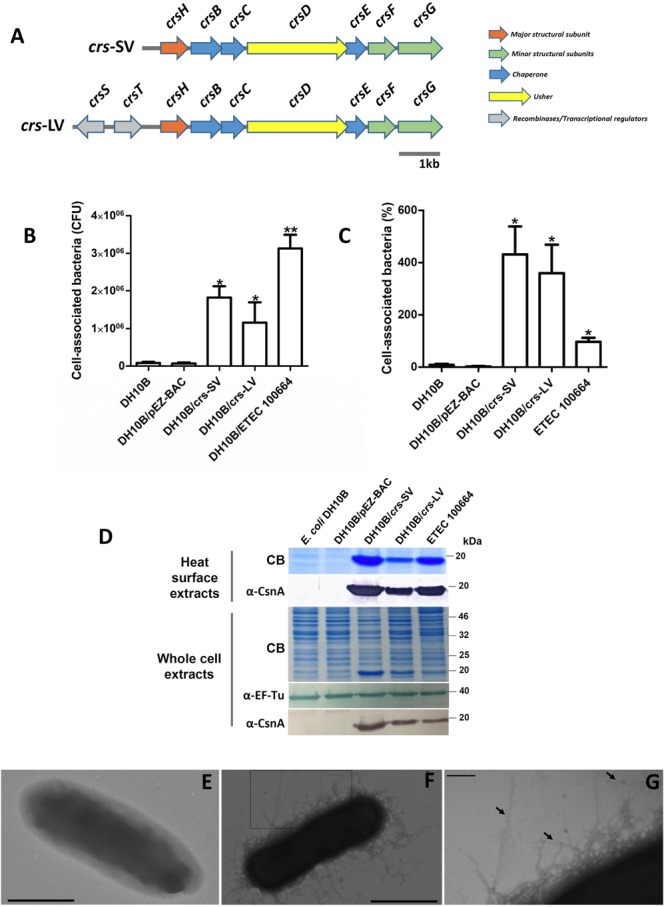
Cloning and expression of *crsHBCDEFG* locus in *E. coli* DH10B. **(A)** Schematic representation of *crsHBCDEFG* locus in its two versions. A short version (*crs*-SV, 7,363 bp) including *crsHBCDEFG* plus 459 bp upstream *crsH* (22 bp at the 3′ end of *crsT*), and the long version (*crs*-LV, 8,761 bp) including *crsSTHBCDEFG* plus 5 bp downstream *crsS*. The *crsH* promoter would be included in both *crs*-SV and *crs*-LV. **(B,C)** Adherence level of *Escherichia coli* DH10B harboring *crs*-SV and *crs*-LV. Host strain without vectors or harboring the empty pEZ-BAC were used as controls. Bars represent cell-associated bacteria in CFU **(B)** or percentage of bacteria associated to the Caco-2 monolayers after 3 h of infection, relative to the initial inoculum **(C)**. Increase in adherence level was significant (^∗^*p* < 0.05, ^∗∗^*p* < 0.001) according to Kruskal–Wallis followed by Dunn’s multiple comparison test. **(D)** Detection of CrsH in surface heat-extracted proteins and whole-cell extracts of recombinant *E. coli* DH10B harboring *crs*-SV and *crs*-LV, subjected to SDS–PAGE. Staining with Coomassie blue and Western blot using anti-CsnA and anti EF-Tu antibodies are shown for each case. **(E–G)** Transmission electron microscopy photographs of negatively stained *E. coli* DH10B/pEZ-BAC **(E)** and *E. coli* DH10B harboring *crs*-SV **(F)**. Bar = 1 μm. **(G)** Increased magnification of the rectangle in panel **(F)**. Arrows indicate pili. Bar = 200 nm.

The *crs* locus encoding CS26 was amplified by PCR. Primers are listed in **Supplementary Table S1**. Two versions of the *crs* locus were generated, a short version (*crs*-SV, 7,363 bp) containing the CU pilus genes (structural subunits, chaperones, and the usher) and a long version (*crs*-LV, 8,761 bp), which also includes two recombinase genes, *crsS* and *crsT* (**Figure 1A**). The primer used to generate *crs*-SV targets a sequence located 459 bp upstream *crsH*, while that used for *crs*-LV targets 5 bp downstream *crsS*. A unique reverse primer was used to obtain both fragments, which targets a sequence 294 bp downstream *crsG*. The *crsH* promoter would be included in both *crs*-SV and *crs*-LV. The *crs*-LV version would include *crsS* and *crsT* promoter regions. PCR products were digested with BamHI, purified, and ligated to previously purified BamHI-digested and dephosphorylated pEZ-BAC (Lucigene). Cloning was checked by PCR (**Supplementary Table S1**) and further by sequencing. Recombinant bacmids were introduced into *E. coli* DH10B or ETEC 100664 by electroporation at 1700V.

### Knocking Out of the *crsHBCDEFG* Locus

Locus *crsHBCDEFG* was removed in ETEC 100664 by allelic replacement as previously described (Datsenko and Wanner, 2000; Sharan et al., 2009; Del Canto et al., 2017). Briefly, the pSIM6 plasmid, encoding the λ Red recombinase system, was introduced by electroporation and transformants were selected in LB agar plates containing ampicillin (Sharan et al., 2009). After 18–20 h, a colony was seeded in a LB broth tube containing ampicillin and incubated at 32°C until reaching OD_600_ = 0.4. The bacterial suspension was incubated at 42°C, with shaking, for 15 min to induce expression of the λ phage Red recombination system genes. Immediately after the induction, cultures were incubated for 10 min on ice and prepared for electroporation to introduce the linear DNA segment including the chloramphenicol acetyltransferase gene (*cat*) flanked by 60-bp regions identical to *crs* locus (60 bp from the start codon of *crsH* and 60 bp at the 3′ end of *crsG*). Transformants were seeded in LB agar plates containing chloramphenicol (30 μg/mL) and incubated at 32°C for 18–20 h. Mutant clones were identified by colony PCR, using a primer that target the *crs* promoter region and a reverse primer that target *cat* (primer G2d-F and Cm-R, **Supplementary Table S1**). The *cat* gene was then removed using the pCP20 plasmid, which encodes the Flp recombinase (Datsenko and Wanner, 2000). In order to evaluate recovery of pili production and cell adherence phenotype, mutant strains were complemented with pEZ-BAC harboring *crs*-SV.

### SDS–PAGE and Western Blotting of Surface Heat-Extracted Proteins

Bacterial surface-associated proteins were obtained to detect the CS26 major structural subunit (CrsH). Overnight cultures (20 mL) in LB were centrifuged at 3,000 × *g* for 10 min, suspended in 100 uL PBS 1X and heated at 60°C for 30 min. The suspension was centrifuged at 3,000 × *g* for 10 min, and the supernatant containing surface proteins was recovered. Proteins were quantified by the Bradford’s method (Bradford, 1976) and 1 μg or 4 μg were subjected to SDS–PAGE (15%). Gels loaded with 4 μg were stained with Coomasie blue, and from those containing 1 μg, proteins were transferred to nitrocellulose membranes to perform CrsH detection by Western blotting. Unspecific protein binding sites were blocked by incubation with 1% bovine serum albumin (BSA) in tris-buffer saline containing 0.05% Tween-20 (BSA/T-TBS), overnight at 4°C. The membrane was further incubated with the rabbit anti-CsnA (CS20 major structural subunit) polyclonal antibody (developed from the purified protein at Genscript, NJ, United States) at a 1:1,000 dilution in BSA/T-TBS for 1 h at room temperature (RT). After three washes with T-TBS, the membrane was incubated with a secondary goat anti-rabbit IgG antibody conjugated to alkaline phosphatase, diluted 1:1,000 in T-TBS, for 1 h at RT. Three washes with T-TBS were performed followed by one with distilled water, and presence of immunoreactive bands was revealed by adding chromogenic substrates nitro-blue tetrazolium and 5-bromo-4-chloro-3′-indolyphosphate.

Detection of CrsH was also performed on whole cell-extracts, in parallel to the detection of the elongation factor Tu (EF-Tu), as loading control. Approximately 4 × 10^8^ bacteria from overnight cultures in LB were boiled at 100°C in Laemmli buffer (Laemmli, 1970) for 10 min and centrifugated at 12,000 × *g* for 5 min. The supernatant was recovered and 10 μL were subjected to SDS–PAGE (15%) into two separate gels. One gel was used for Coomassie staining and the other for Western blot detections. Detection of CrsH was performed as described above. For detection of EF-Tu, the same procedure was followed but a commercially available anti EF-Tu monoclonal antibody (Hycult Biotech, Wayne, PA, Unites States) was used in a 1:2,000 dilution followed by incubation with an anti-mouse IgG conjugated to alkaline phosphatase in a 1:1,000 dilution. Immunoreactive bands were identified by adding a mix the substrates nitro-blue tetrazolium chloride and 5-bromo-4-chloro-3′-indolyphosphate p-toluidine salt for chromogenic detection.

### Transmission Electron Microscopy (TEM) and Immunogold Staining

Drops of 10 μL of overnight cultures in LB were incubated over carbon-formvar coated nickel grids, for 5 min at 37°C. After three washes with PBS 1X, bacteria were fixed with 2.0% glutaraldehyde and negatively stained with 0.5% phosphotungstic acid. To determine if anti-CsnA polyclonal antibodies recognize CS26, immunogold staining was performed. Washed grids containing bacteria were incubated in 1% BSA – 0.01 M glycine in PBS, for 1 h at RT, and then with the anti-CsnA polyclonal antibody in a 1:100 dilution in 0.2% BSA-0.05% Tween 20 (T-BSA), for 1 h at RT. After three washes with (T-BSA), grids were incubated with the secondary goat anti-rabbit IgG conjugated to 10nm-gold particles, in a 1:10 dilution. Three washes with T-BSA were performed and bacteria were fixed and negatively stained as described above. Samples were analyzed at 80 kV at the Center for the Development of Nanoscience and Nanotechnology, Universidad de Santiago de Chile, with an Hitachi HT7700 transmission electron microscope; or at the Laboratory of Electron Microscopy, Facultad de Ciencias Biológicas, Pontificia Universidad Católica de Chile, with a Philips Tecnai 12 microscope.

### Cell Adherence Assays

Adherence assays were performed on Caco-2 cell monolayers. Cells were kept and subcultured in Dulbecco’s modified Eagle Medium (DMEM) containing 10% bovine fetal serum and 1% antibiotics/antifungals (penicillin, streptomycin, and amphotericin B), at 37°C in an incubator with an atmosphere containing 95% air and 5% CO_2_. After reaching 100% confluence (7 days), cells were infected at a multiplicity of infection of 10 bacteria per cell. Approximately 3.5 × 10^6^ CFU, estimated by measuring OD600, were added and the plate was kept in the cell culture incubator at 37°C for 3 h. Planktonic bacteria were removed and cells were washed five times with PBS 1X. To recover cell-associated bacteria, cell layers were lysed by adding 0.1% Triton X-100 in PBS 1X. The number of viable bacteria in the initial inoculum and in the cell-associated fraction was determined by seeding in LB agar plates and colony counting by the microdrop method (Pfeltz et al., 2001). Results were expressed as cell associated CFU or percentage of cell associated bacteria relative to the initial inoculum. In order to asses the influence of type 1 pilus on observed bacterial adherence capacity, assays were performed in presence of 1% of D-mannose.

### Screening of CS26 and γ2-CF Genes in Databases and Phylogenetic Tree

To determine the distribution of CS26, and other γ_2_-CF (CS12, CS18, CS20, and CS30), among ETEC strains, a search for the genes encoding the major structural subunit was performed in the *E. coli* genomes available in the NCBI Assembly RefSeq database (accessed June, 2018) using Large Scale Blast Score Ratio (LS-BSR) with tblastn (Sahl et al., 2014). To predict the diarrheagenic pathotype of the positive strains, marker genes were included in the screening. A list of the genes and genomes included in the analysis, and their accession codes, is shown in **Supplementary Table S2**. BSR values in the matrix were represented as a color map using the gplots package of R (R Core Team, 2014; Warnes et al., 2016). A phylogenetic tree based on 30,465 single nucleotide polymorphisms (SNPs) in the core genome was built with γ_2_-CF positive records using kSNP v3.1 with the parsimony method and 100 bootstrap replicates (Gardner et al., 2015). The tree was drawn with the Interactive Tree of Life v2 online software (Letunic and Bork, 2011). Phylogroups were determined according to the scheme proposed by Clermont (Clermont et al., 2011, 2013), by screening the primers with LS-BSR using blastn. Serotypes and sequence types were determined using the Serotype finder and MLST 2.0 tools available in the Center for Genomic Epidemiology (Jaureguy et al., 2008; Joensen et al., 2015).

### Statistical Analysis

At least three independent cell adherence assays were performed and data was analyzed using the Kruskal–Wallis test followed by Dunn’s multiple comparison. Differences were considered as significant if *p* < 0.05.

## Results

### Detection of CS26 and Its Role in Bacterial Adherence

The locus *crsHBCDEFG* [*crs*-short version, (*crs*-SV)] was amplified from ETEC 100664, a strain obtained during the Global Enterics Multicenter Study (GEMS) (Kotloff et al., 2013; Del Canto et al., 2017), cloned in pEZ-BAC and introduced into *E. coli* DH10B, in order to determine if it confers adherence capacity. In addition, a longer version of the locus was also cloned [*crs*-long version, (*crs*-LV)], as two genes encoding putative recombinases (*crsS* and *crsT*) were identified in the ETEC 100664 genome, upstream *crsH* (**Figure 1A**). It is known that these kinds of proteins act as transcriptional regulators, allowing promoter inversion and phase variation of CS18 and the type 1 fimbria (Klemm, 1986; Honarvar et al., 2003). *E. coli* DH10B harboring either of the two versions displayed a significantly higher adherence capacity (*p* < 0.05) to intestinal Caco-2 cells compared to the control without vectors or harboring the empty bacmid, and this was also observed in the case of ETEC 100664 (**Figures 1B,C**). For both recombinant DH10B clones harboring *crs*, adherence percentages indicate that the number of cell-associated bacteria after 3 h is higher than the initial inoculum (**Figure 1C**). As bacterial duplication is expected to occur in about 20 min, we can estimate that cell-associated bacteria are about 22% of the total bacterial population (cell associated and planktonic), after 3 h of infection. The number of cell-associated CFU in the case of ETEC 100664 was significantly higher compared to the DH10B/*crs*-LV only, but when results were expressed as percentages, there were no significant differences between ETEC 1000664 and the recombinant clones harboring *crs*.

Heat extracted surface proteins were analyzed by SDS–PAGE and Coomassie blue staining. A band of about 20 kDa, which corresponds to the mature CrsH molecular weight (178aa, predicted molecular weight 18 kDa, lacking a 21aa-signal peptide required for secretion to the periplasm) was observed in extracts of both recombinant clones and ETEC 100664, but not in the host DH10B or the same strain harboring the empty bacmid (**Figure 1D**). Western blot using an anti-CsnA polyclonal antibody allowed detection of this band, indicating cross-reaction with CrsH. Alignment of the amino acid sequences of mature CsnA and CrsH indicated they share 57% identity and 71% similarity. These results were also observed when whole-cell extracts were analyzed. The CrsH band recognized in both extracts of DH10B/*crs*-SV seems to be denser compared to the bands observed in DH10B/*crs*-LV and ETEC 100664, which suggests that it produces higher amounts of fimbriae.

Analysis by TEM of *E. coli* DH10B/*crs*-SV allowed observation of rigid fibers, of between 7 and 11 nm of width, evenly distributed at the bacterial surface. These structures were not observed in *E. coli* DH10B harboring the empty pEZ-BAC bacmid. Therefore, this data indicates that the locus *crsHBCDEFG* encodes a functional pilus, with the capacity to confer adherence to epithelial cells (**Figures 1E–G**).

**FIGURE 2:**
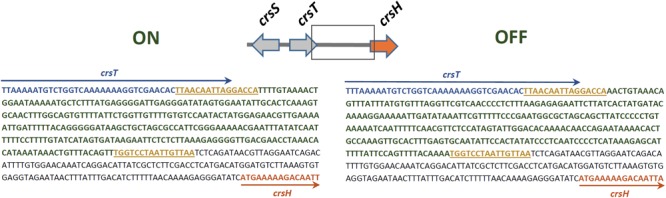
Sequence of the *crsHBCDEFG* promoter region in its two orientations (ON and OFF), according to CrsH production and adherence capacity. Sequences in blue and orange fonts represent segments of *crsT* and *crsH*, respectively, delimited by arrows of the same color. The segment in blue fonts represents the invertible sequence and the flanking 16-bp sequences in yellow represent inverted repeats.

Sequencing and comparison of the putative *crsHBCDEFG* promoter region in representative clones of *E. coli* DH10B harboring *crs*-SV or *crs*-LV, along with analysis of ETEC 100664 draft genome, allowed identification of a reversible 279 bp segment, which is flanked by 16 bp-inverted repeated sequences. Given that adherence capacity and production of CrsH was directed by *crs*-SV, which lacks the recombinase genes and therefore, could not be inverted, we assumed that the orientation of the invertible segment found in this case corresponds to the “ON-state” (**Figure 2**). The “OFF” state, found in a colony of DH10B/*crs*-LV, would correspond to the inverted orientation of this segment. Segments in both orientations were found in the ETEC 100664 draft genome (ON state: contig 7180000013456, NCBI nucleotide accession NZ_LGMS01000085.1; OFF state: contig 7180000013431, NZ_LGMS01000110.1).

**FIGURE 3:**
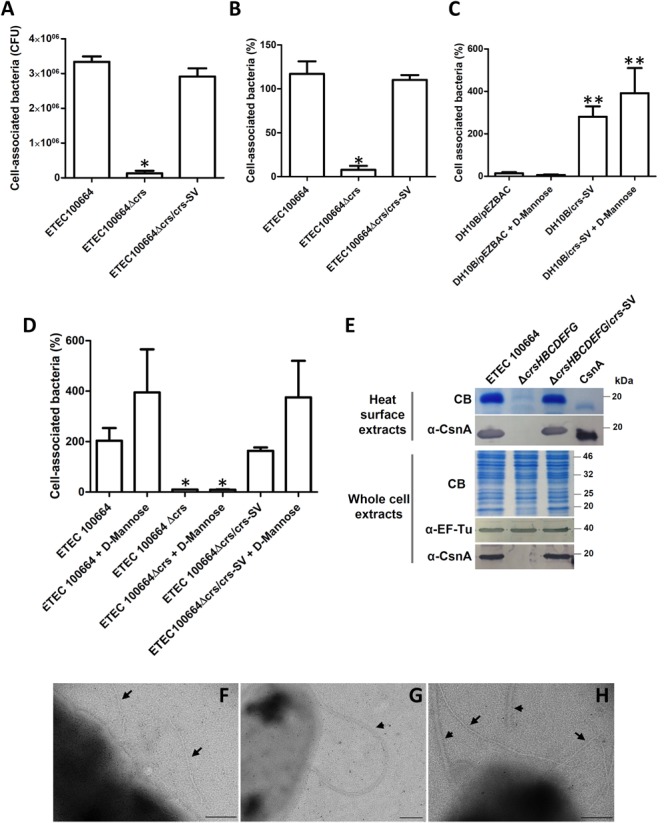
Functionality of *crsHBCDEFG* in ETEC100664. **(A,B)** Adhesion level of ETEC100664 to Caco-2 cells, after knocking out and *in trans* complementation of *crsHBCDEFG*, expressed as cell-associated bacteria in CFU **(A)** or percentage of bacteria associated to the Caco-2 monolayers, relative to the initial inoculum **(B)**. ^∗^Decrease in adherence level was significant (*p* < 0.05) according to Kruskal–Wallis followed by Dunn’s multiple comparison test. **(C,D)** Adhesion percentages, relative to the initial inoculums, in presence of 1% D-mannose, to asses the influence of type 1 pilus. ^∗∗^Adherence level was significantly higher compared to DH10B/pEZ-BAC, regardless the presence of D-mannose. ^∗^Adherence level was significantly lower (*p* < 0.05), compared to the wild type and complemented strain, regardless presence of D-mannose. **(E)** Production of CrsH in the ETEC 100664 mutant and complemented derivative strains. CrsH was recognized by Coomassie blue staining and by Western blot, using polyclonal anti-CsnA antibodies, in surface heat-extracted proteins and whole-cell extracts. EF-Tu was also detected by Western blot as loading control. **(F–H)** Transmission electron microscopy (TEM) analysis of negatively stained ETEC 100664 **(F)** and its mutant **(G)** and complemented **(H)** derivative strains. Arrows indicate pili and arrowheads indicate flagella. Bars = 200 nm.

Functional evaluation of *crsHBCDEFG* on ETEC100664 was consistent with results obtained with recombinant *E. coli* DH10B. A significant decrease (*p* < 0.05) in the adherence capacity to Caco-2 cells was observed in comparison with the wild type strain, which was reverted after *in trans* complementation with *crs*-SV (**Figures 3A,B**). This was observed by expressing results as cell-associated CFU or percentage relative to the initial inoculum. Assays performed with 30 min of infection showed a similar tendency (data not shown). Furthermore, adherence of bacteria producing CS26 in our assays does not seem to be determined by type 1 pilus, as presence of 1% of D-mannose during the infection did not cause any significative effect (**Figures 3C,D**).

The tendency found in adherence level of ETEC 100664 and the ETEC 100664 *crsHBCDEFG* mutant was in line with the presence of the CrsH protein band in heat-extracted surface proteins or whole-cell extracts separated by SDS–PAGE, supporting the role of CS26 (**Figure 3E**). The anti-CsnA antibody also recognized the CrsH band produced by the complemented mutant strain (**Figure 3E**). Analysis by TEM of negatively stained bacteria allowed observation of pili with similar features to those observed in the recombinant DH10B/*crs*-SV strain, in ETEC 100664 (**Figure 3F**). However, in most of the observed bacteria, pili was not so abundant as evidenced in the recombinant strain. This kind of structures were not observed in the ETEC100664 *crsHBCDEFG* mutant strain (**Figure 3G**), but they were present in the mutant complemented with the *crs*-SV, in a similar fashion to that seen in the wild type strain (**Figure 3H**).

**FIGURE 4:**
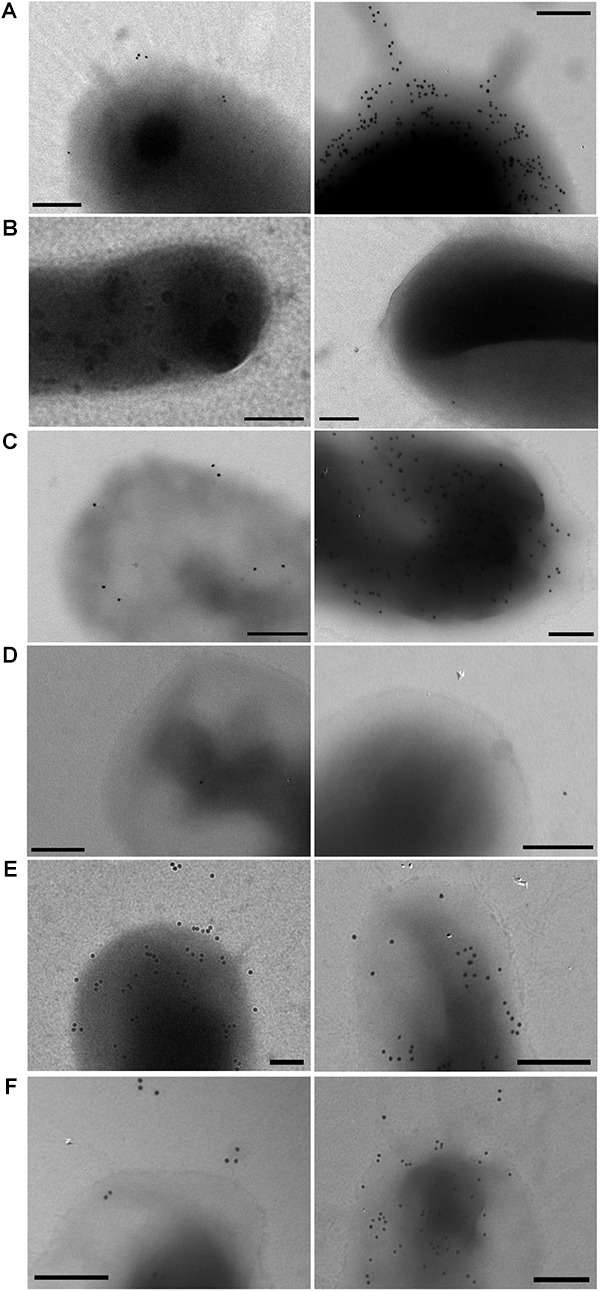
Immunogold labeling for detection of CrsH. **(A)** ETEC 100664; **(B)** ETEC 100664Δ*crs*; **(C)** ETEC 100664Δ*crs*/*crs*-SV; **(D)** *E. coli* DH10B/pEZ-BAC; **(E)** *E. coli* DH10B/*crs*-SV; and **(F)** *E. coli* DH10B/*crs*-LV. Presence of CrsH was detected in non-permeabilized bacteria using an anti-CsnA antibody. Two bacteria are shown for each strain to show variability in labeling patterns in the case of ETEC 100664, ETEC 100664Δ *crsHBCDEFG* mutant complemented strain and DH10B/*crs*-LV. Bars = 200nm.

The cross reaction of anti-CsnA with CrsH was also evident by immunogold labeling of non-permeabilized bacteria (**Figure 4**). Gold particles decorating ETEC100664 surface were not observed on the ETEC100664 *crsHBCDEFG* mutant, but they were detected in the mutant strain complemented with *crs*-SV. In addition, immunoreactivity was also evident in both recombinant strains DH10B/*crs*-SV and DH10B/*crs*-LV, but not in the control host strain harboring the empty bacmid. In those strains that displayed surface immunoreactivity, the abundance of gold particles was variable, with some bacteria decorated with 10 – 20 particles and others with more than 100 particles. This behavior was not clearly evident in DH10B/*crs*-SV, in which the staining degree seemed to be less variable. These results suggest that production of CS26 is being regulated by CrsS and CrsT, whose genes are harbored by DH10B/*crs*-LV, ETEC10664 and the ETEC10664 *crsHBCDEFG* mutant, but are absent in DH10B/*crs*-SV. However, given that *crs*-SV harbors the reversible segment in the region upstream *crsH*, regulation by phase variation could occur if regulators are present. That would be the case of the complemented ETEC10664 *crsHBCDEFG* mutant strain.

### Genome-Based Analysis of ETEC Strains Harboring CS26

**FIGURE 5:**
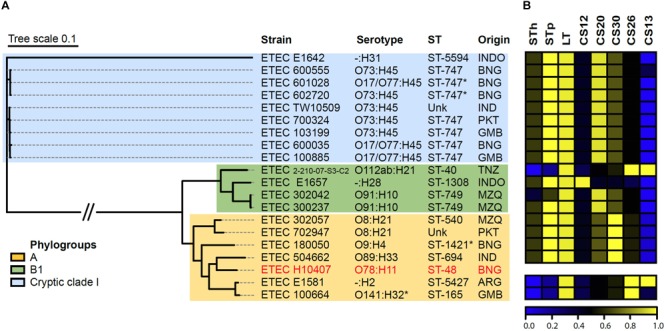
Genetic analysis of CS26+ strains, in comparison to other ETEC strains harboring γ_2_-CFs. **(A)** Parsimony tree based on core genome SNPs of γ_2_-CFs ETEC strains, including serotypes, sequence types, geographic origin, and phylogroups. Prototype ETEC H10407 was included as reference. INDO: Indonesia, BNG: Bangladesh, IND: India, PKT: Pakistan, GMB: The Gambia, TNZ: Tanzania, MZQ: Mozambique, and ARG: Argentina. ^∗^O141:H32, Serotype Finder did not recognize O serogroup for this strain, but it was recognized by the agglutination method (Del Canto et al., 2017). ^∗^ST-747 and ^∗^ST-1421, presumptive sequence types. Unk: unknown sequence type. **(B)** Color map representing presence of toxins and CF genes on γ_2_-CFs+ ETEC records in NCBI assembly Refseq database. The order of strains matches the order in the phylogenetic tree. Color scale represents blast score ratio (BSR) values. A value of 1 indicates presence of a gene encoding an identical protein. CS13 was the only non-CF detected.

In order to find and characterize genomes of strains harboring CS26, in comparison with strains harboring other γ_2_-CFs, a screening of genes encoding major structural subunits of γ_2_-CFs was performed on the *E. coli* genomes available in the NCBI assembly RefSeq databank. To identify ETEC strains, genes encoding STh, STp, LT, and CFs major structural subunits, were included in the screening. A record was considered as an ETEC genome if at least one toxin gene was detected. Two CS26+ ETEC genomes besides ETEC100664 were found, which, unlike this one, were both positive for CS13 (**Figure 5**). The three CS26+ strains were LT+ only. Among the γ_2_-CFs positive records, one was positive for CS12, 11 for CS20 and four for CS30. All of them, except one CS20+, were positive for STp and LT genes (**Figure 5B**). No other known CFs genes were found in this set of genomes. According to a core SNP-based phylogenetic tree, these γ_2_-CFs+ records represent a genetically diverse group, with strains isolated in eight countries from three continents (Asia, Africa, and South America) (**Figure 5A**). CS26+ strains belong to phylogroups A and B1, including three different serotypes (O141:H32, O112ab:H21, and -:H2) and sequence types (ST-165, ST-5427, and ST-40). Records positive for other γ_2_-CFs also represent phylogroups A and B1, but also the “cryptic” clade I lineage, particularly for CS20+, in which serotype O73:H45 and sequence type ST-747 were predominant.

## Discussion

More than twenty diverse CFs have been discovered and described in human ETEC strains (Gomes et al., 2016; von Mentzer et al., 2017). Given that many isolates worldwide have been negative for the detection of known CFs, it was expected that discovery of loci encoding putative CFs, mainly based on DNA sequence analysis, increase that number and contribute to complete the picture of the highly diverse ETEC adhesins repertoire. Here, we found that CS26, encoded by *crsHBCDEFG*, is actually a fimbria conferring adherence capacity to a clinical ETEC isolate (ETEC 100664) and to a laboratory non-pathogenic strain.

CS26 was recognized as a pili of 7–10 nm in diameter, similar to other γ_2_-CFs (Tacket et al., 1987; Viboud et al., 1993; Valvatne et al., 1996; von Mentzer et al., 2017). As reported for CS20, thin and thicker structures were simultaneously observed by TEM analysis of negatively stained bacteria, likely representing unwound and wound pili confirmations (Singh et al., 2015). Cross reaction of anti-CsnA antibodies with CrsH was evidenced by Western blot. Although antibody cross- reactivity is expected between proteins sharing more than 50% identity, it had not been determined if there are antibody cross-reactions between γ_2_-CFs of human ETEC. Western blot detection of CrsH, in parallel to EF-Tu, suggests that DH10B/*crs*-SV would produce more pili than DH10B/*crs*-LV and ETEC 100664. This coincides with absence of putative regulators CrsS and CrsT in DH10B/*crs*-SV and, in turn, absence of regulation by phase variation. Results of immunogold are consistent with Western blot results. In all the strains harboring *crsS* and *crsT*, the amount of gold particles associated to bacterial surface was variable, ranging from 10 to 20 to more than a hundred per bacterium. In contrast, the abundance of gold particles in DH10B/*crs*-SV, which lacks *crsS* and *crsT* seemed to be more evenly distributed across the sample. Further research will be required to accurately describe the regulation of CS26 production by phase variation.

In our experiments, for most of the cases, the pattern of labeling observed in immunogold staining does not resemble the shape of a pilus. This could lead to the interpretation of a negative result, particularly if there are few particles per bacterium. However, a previous report for detection of CS20 using an anti-CsnA polyclonal antibody, developed from the purified protein, showed a similar labeling pattern even when expression of the pilus was induced (Valvatne et al., 1996). Labeling with gold particles following the shape of the pili could have been obtained if purified structures had been used to develop the antibodies. That was the case for previous detection of γ_2_-CFs CS12 and CS18 (Tacket et al., 1987; Viboud et al., 1993).

Differences noticed in detection by immunoassays were not significantly reflected in evaluation of adherence capacity. This could be explained by the fact that infections were performed for 3 h, a period in which bacterial populations could growth to saturate available bacterial binding sites in Caco-2 cells. In addition, we can speculate that in our *in vitro* infection assays, bacteria producing a reduced number of pili could be capable to attach cell surface just like bacteria producing a higher number of structures do it. A significant difference in adherence capacity was noticed between ETEC 100664 and DH10B/*crs*-LV when results were expressed as cell associated CFU, but the difference does not seem to be dependent on the level of CrsH or pili production. Given that there were no significant differences between ETEC 100664 and DH10B/*crs*-LV when results were expressed as percentage of cell-associated bacteria, relative to the initial inoculum, we speculate that the difference could depend on the replication rate or bacterial viability. In this scenario, we noticed that CFU counts of DH10B/*crs*-SV and DH10B/*crs*-LV were lower than those of DH10B harboring the empty bacmid, when seeded from suspensions having equal OD600’s. This suggests that recombinant expression of fimbrial genes and subsequent production of fimbriae might affect bacterial viability, particularly in this case, in which replication of the bacmid harboring loci was induced up to 50 copies per cell (according to pEZ-BAC manufacturer indications).

The recent finding of CS30 and several loci encoding putative γ_2_-CFs (Del Canto et al., 2017; Sahl et al., 2017; von Mentzer et al., 2017), suggests that these structures may be more diverse and common among ETEC than initially thought. In addition, in this context, ETEC strains harboring the *cma* locus, which encodes the putative γ_2_-CF CS27b, were found to be emerging agents of infectious diarrhea in Bangladesh (Begum et al., 2018). Therefore, despite the fact of a lower prevalence compared to the most common ETEC adhesive structures (CFA/I, CS6, CS21 or EtpA), it would be appropriate to consider γ_2_-CFs for future design of vaccine candidates. In this scenario, it will be relevant to identify the most common representatives and to determine the potential cross-antigenicity between ETEC γ_2_-CFs, as has been reported between some α-CFs (CFA/I, CS1, CS2, CS4, CS5, CS7, CS14, and CS17) in different combinations (McConnell et al., 1989; Rudin et al., 1996; Qadri et al., 2006). Based on the amino acid identity between mature major structural subunits of γ_2_-CFs (CS12, CS18, CS20, CS30, and CS26), which ranges between 50 and 77%, we hypothesize that cross-reactivity should occur.

Genome-based characterization of ETEC harboring γ_2_-CFs will further contribute to elucidate their cross-sectional features. Currently, the low detection frequency among ETEC causing diarrhea in humans, is paralleled by a limited number of records representing γ_2_-CF+ strains in genome databanks. We found that ETEC strains harboring CS26, CS20, and CS30 are diverse, including several serotypes and sequence types from phylogroups A and B1, but also from the cryptic *E. coli* clade I (particularly CS20+ strains). Presence of ETEC in this cryptic clade has been reported, but not analyzed in depth (Walk, 2015). Overall, this supports the claim that the acquisition of clusters encoding γ_2_-CFs by ETEC strains has occurred by multiple horizontal transfer events. Further research including an increased number of representative strains will help to define their phylogenetic/phylogenomic features with more accuracy.

The *crsH* gene sequence found in ETEC 100664 shares 91% identity with the first reported partial *crsH* sequence, derived from the ETEC strain MH2416 (Nucleotide NCBI accession HQ203050.1) (Nada et al., 2011; Del Canto et al., 2017). Assuming that it could represent a genetic variant, it was named *crsH_b_*. However, the two additional CS26+ strains found in this work, by bioinformatic screening, harbor sequences identical to the ETEC 100664 *crsH*. Alignment of nucleotide sequences indicate that discrepancies in *crsH* segment of ETEC MH2416 (449 nt) reside mostly in its 3′ end, while the segment between positions 4-393 is identical to *crsH* found in the other ETEC strains. On the other hand, as strain MH2416 was collected in a study carried out between 2000 and 2002 in Egypt, and ETEC 100664 was collected between 2007 and 2011 in The Gambia, there could be a divergence by separate evolution. Sequencing of more CS26+ strains will help to clarify if there are *crsH* variants or not.

Among CS26+ ETEC, the co-ocurrence with CS13, a κ-CU pilus, was the only detected with CFs from other families. The association with CS13 had been reported for CS26+ strains and also for CS27+, CS28+, and CS30+ strains (Nada et al., 2011; von Mentzer et al., 2017). Furthermore, genetic variants of the *aal* locus, which encodes CS23 (other κ-CF), were found in CS26+ and CS28+ strains, including ETEC 100664 (Del Canto et al., 2017). This suggests that κ-CFs might be frequent among ETEC producing γ_2_-CFs. On the other hand, a common factor for this group is the association with LT-only or LT-STp profiles, but not with STh. In general, ST-producing ETEC, alone or in combination with LT, have been significantly associated with severe and moderate-to-severe diarrhea cases (Kotloff et al., 2013). Given that STh is more commonly produced by human ETEC than STp, this association might be mainly attributed to that variant. In contrast, LT-producing ETEC have been found in similar proportions among diarrhea cases and control individuals without diarrhea (Rivera et al., 2010; Liu et al., 2014). However, given that ETEC producing LT-only and STp-LT are also causing diarrhea worldwide (in fact, at least 17 of the 19 genome records analyzed in our study correspond to ETEC strains obtained from diarrhea cases) it will be worth to gain more insights in the biology and epidemiology of these strains.

Given that in all the known γ_2_-CFs of human ETEC, the major structural subunit is named with a combination of three letters plus an A (CswA of CS12; FotA of CS18; CsnA of CS20; and CsmA of CS30) (Madhavan and Sakellaris, 2015; Del Canto et al., 2017; von Mentzer et al., 2017), we propose to rename the CS26 structural major subunit as CrsA, and its gene as *crsA*.

## Conclusion

CS26 is a functional pilus capable of conferring adherence capacity to ETEC strains and to a laboratory non-pathogenic *E. coli* strain. This adds another representative to the list of functionally evaluated CFs of human ETEC. In addition, the cross reaction between CS26 major structural subunit CrsA (formerly known as CrsH) with anti-CsnA antibodies suggest presence of common epitopes among the growing family of γ_2_-CFs, which could determine a cross-blocking effect of antibodies on bacterial adherence.

## Author Contributions

LC and AT contributed to experimental work, data analysis, and wrote the manuscript. RVa, GV, and DG contributed to experimental work and data analysis. ML contributed to study the design and data analysis. DM contributed to experimental work and genome analysis. MO’R contributed to the study design, data analysis, and reviewed the manuscript. DR contributed to genome analysis and reviewed the manuscript. OS contributed to the study design, genome analysis, and reviewed the manuscript. RVi contributed to the study design and reviewed the manuscript. FDC contributed to genome analysis, data analysis, and wrote the manuscript.

## Conflict of Interest Statement

The authors declare that the research was conducted in the absence of any commercial or financial relationships that could be construed as a potential conflict of interest.
