# Supplementary material for: Coli Surface Antigen 26 Acts as an Adherence Determinant of Enterotoxigenic Escherichia coli and Is Cross-Recognized by Anti-CS20 Antibodies
Source: Front Microbiol. 2018 Oct 16;9:2463. doi: 10.3389/fmicb.2018.02463 (PMC6232838; doi:10.3389/fmicb.2018.02463)
Supplement: Supplementary file 1 [file Table_1.docx]

**Table S1**. Primers used in this study.

| **Primer** | **Sequence (5’ to 3’)** | **Use** | **Reference** |
| --- | --- | --- | --- |
| **G2d-F0** | GATTTTTAAGACGTTACATCCTGAG | Cloning of *crs* locus (*crs*-LV) | This work |
| **G2d-F** | AGGTCGAACACTTAACAATTAGG | Cloning of *crs* locus (*crs*-SV) | Del Canto et al., 2015 |
| **G2d-R** | AACAGAAGCCACTGGAGCAC | Cloning of *crs* locus (*crs*-SV and *crs*-LV) | This work |
| **G2d-checkR** | GAGGCAGCAGCAGGATCAG | Confirmation of cloning of *crs* locus | This work |
| **G2c-CmF** | ATGAAAAAGACAATTATGTCTCTGGCTGTGGTTTCAGCTTGTGTAGGCTGGAGCTGCTTC | Mutagenesis of *crs* | Del Canto et al., 2015 |
| **G2c-CmR** | CTAGTTCTCCCATCGGGAGATTATTTTTATTGATACATTACATATGAATATCCTCCTTAG | Mutagenesis of *crs* | Del Canto et al., 2015 |
| **CmR** | ATGAAAGACGGTGAGCTGGT | Confirmation of mutagenesis | Del Canto et al., 2015 |
| **BEZ-F1** | CACTTTATGCTTCCGGCTCGTATG | Confirmation of cloning of *crs* locus | Lucigene |
| **BEZ-R1** | GGGATGTGCTGCAAGGCGATTAAG | Confirmation of cloning of *crs* locus | Lucigene |

**Table S2**. Accession codes of sequences used in this study.

| **Locus/Genome** | **Description** | **NCBI Database** | **Accession code (region)** |
| --- | --- | --- | --- |
| *crsSTHBCDEFG* | Locus encoding CS26 | Nucleotide | NZ_LGMS01000110.1 (31...8492) |
| STh | Human heat-stable enterotoxin | Nucleotide | NC_009786.1 (11352..11570) |
| STp | Porcine heat-stable enterotoxin | Nucleotide | FN649417 (57051..57269) |
| LT | Heat labile enterotoxin subunit B | Nucleotide | FN649417.1 (50223..50597) |
| CFA/I | Major structural subunit gene (MjSS) *cfaB* | Nucleotide | M55661.1 (1593..2105) |
| CS1 | MjSS gene *cooB* | Nucleotide | LN870269.1 (1..594) |
| CS2 | MjSS gene *cotA* | Nucleotide | Z47800.1 (1255..1767) |
| CS3 | MjSS gene *cstA* | Nucleotide | X16944.1 (4153..4659) |
| CS4 | MjSS gene *csaB* | Nucleotide | AF296132.1 (1028..1531) |
| CS5 | MjSS gene *csfA* | Nucleotide | AJ224079.2 (1427..2038) |
| CS6 | MjSS gene *cssA* | Nucleotide | GQ241334.1 (1..465) |
| CS7 | MjSS gene *csvA* | Nucleotide | AY009095.1 (144..755) |
| CS8 | MjSS gene *cofA* | Nucleotide | EU107088.1 (1..717) |
| CS12 | MjSS gene *cswA* | Nucleotide | AY009096.1 (3505..4095) |
| CS13 | MjSS gene *cshE* | Nucleotide | X71971.1 (4734..5510) |
| CS14 | MjSS gene *csuA1* | Nucleotide | AY283611.1 (1287..1796) |
| CS15 | MjSS gene *nfaA* | Nucleotide | X64623.1 (136..636) |
| CS17 | MjSS gene *csbA* | Nucleotide | AY515609.1 (1276..1782) |
| CS18 | MjSS gene *fotA* | Nucleotide | AF335469.1 (2044..2661) |
| CS19 | MjSS gene *csdA* | Nucleotide | AY288101.1 (1031..1531) |
| CS20 | MjSS gene *csnA* | Nucleotide | AF438155.1 (1..588) |
| CS21 | MjSS gene *lngA* | Nucleotide | EU107107.1 (1..621) |
| CS22 | MjSS gene *cseA* | Nucleotide | AF145205.1 (66..566) |
| CS23 | MjSS gene *aalE* | Nucleotide | JQ434477.1 (5417..6214) |
| CS30 | MjSS gene *csmA* | Nucleotide | LT174529.1 (36760..37365) |
| CS26 | MjSS gene *crsA* | Nucleotide | NZ_LGMS01000110.1 (1883..2482) |
| ETEC 100664 | CS26+ ETEC genome | Assembly Refseq | GCF_001911805.1 |
| ETEC E1581 | CS26+ ETEC genome | Assembly Refseq | GCF_002207345.1 |
| ETEC  2-210-07-S3-C2 | CS26+ ETEC genome | Assembly Refseq | GCF_000703945.1 |
| ETEC E1657 | CS12+ ETEC genome | Assembly Refseq | GCF_002206765.1 |
| ETEC 602720 | CS20+ ETEC genome | Assembly Refseq | GCF_001911145.1 |
| ETEC 601028 | CS20+ ETEC genome | Assembly Refseq | GCF_001912525.1 |
| ETEC 103199 | CS20+ ETEC genome | Assembly Refseq | GCF_001911405.1 |
| ETEC TW10509 | CS20+ ETEC genome | Assembly Refseq | GCF_000190995.1 |
| ETEC 302042 | CS20+ ETEC genome | Assembly Refseq | GCF_001910825.1 |
| ETEC 600555 | CS20+ ETEC genome | Assembly Refseq | GCF_001912235.1 |
| ETEC 100885 | CS20+ ETEC genome | Assembly Refseq | GCF_001911065.1 |
| ETEC 600035 | CS20+ ETEC genome | Assembly Refseq | GCF_001910995.1 |
| ETEC E1642 | CS20+ ETEC genome | Assembly Refseq | GCF_002207125.1 |
| ETEC 700324 | CS20+ ETEC genome | Assembly Refseq | GCF_001912565.1 |
| ETEC 300237 | CS20+ ETEC genome | Assembly Refseq | GCF_001910915.1 |
| ETEC 180050 | CS30+ ETEC genome | Assembly Refseq | GCF_000356045.2 |
| ETEC 702947 | CS30+ ETEC genome | Assembly Refseq | GCF_001912455.1 |
| ETEC 504662 | CS30+ ETEC genome | Assembly Refseq | GCF_001911225.1 |
| ETEC 302057 | CS30+ ETEC genome | Assembly Refseq | GCF_001911075.1 |
